# Auditing improvements to physical health in the acute psychiatric inpatient setting

**DOI:** 10.1192/bjo.2021.308

**Published:** 2021-06-18

**Authors:** Joshua A. Silverblatt, Risha Ruparelia, Ayotunde Shodunke

**Affiliations:** 1 Watford General Hospital; 2 Kingfisher Court

## Abstract

**Aims:**

Whilst patient psychiatric health is the primary focus in the acute psychiatric inpatient setting, there has been a recent focus on ensuring a greater integration with physical health to address the physical health outcome inequalities between patients with psychiatric conditions and those without. Despite the ward having a robust physical health clerking proforma, there were issues with its completion; at initial clerking patients often aren't able, or refuse, to consent to physical examination or investigations. This lead to the trust's electronic physical health form, designed to collate these results, not always being completed. Our aim was to increase the rates of completion.

**Method:**

Changes to ward handover sheets were made in an effort to increase rates of physical health form completion and improve 24 and 72 hour completion rates. Columns were added delineating which parts of the physical clerking were outstanding, ensuring the MDT were aware of which jobs needed actioning. Data for two months prior and post intervention were analysed.

**Result:**

266 admissions were analysed for the two months prior and post the intervention. Form completion rose from July (88%) to October (100%), with 24 and 72 hour completion rate increasing from 47% & 55% respectively, to 84% & 96%, during the same time period. Greater completion rates of physical health forms led to increased knowledge of patients’ physical health issues. Having 96% of patients physical health issues within three days of admission (cf. 55%, July), led to a 'physical health huddle' being held during the MDT. This provided a platform to discuss relevant physical health treatment plans with the whole team. These findings were summarised under a new column on the handover sheet and updated biweekly during the MDT meeting. Placement on the handover sheet ensured daily visibility to all staff.

**Conclusion:**

Simple structural changes can bring physical health to the fore in psychiatric care. Timely and more complete physical health data enabled biweekly reviews of physical health issues and allowed input across the MDT. Increased knowledge and awareness of physical health issues led to an increase in medical review requests. These are currently performed on an ad hoc basis, which can be quite disorganised and inefficient. The results above, of improved physical health outcomes based on a structured approach, have led to a recommendation of a biweekly physical health clinic, with specific staffing allocation, to ensure a more thorough and efficient way to address physical health.

